# A Facile Solvent Free Claisen-Schmidt Reaction: Synthesis of α,α′-*bis*-(Substituted-benzylidene)cycloalkanones and α,α′-*bis*-(Substituted-alkylidene)cycloalkanones

**DOI:** 10.3390/molecules17010571

**Published:** 2012-01-09

**Authors:** A. F. M. Motiur Rahman, Roushown Ali, Yurngdong Jahng, Adnan A. Kadi

**Affiliations:** 1 Department of Pharmaceutical Chemistry, College of Pharmacy, King Saud University, Riyadh11451, Saudi Arabia; 2 College of Pharmacy, Yeungnam University, Gyeongsan 712-749, Korea; 3 Department of Chemistry, College of Science, King Saud University, Riyadh 11451, Saudi Arabia

**Keywords:** Claisen-Schmidt reaction, crossed-aldol reaction, cycloalkanone, solvent free

## Abstract

Solvent-free Claisen-Schmidt reactions of cycloalkanones with various substituted benzaldehydes (aryl aldehydes) using solid NaOH (20 mol%) and applying a grinding technique were studied. Quantitative yields (96–98%) of α,α'-*bis*-(substituted-benzylidene)cycloalkanones were obtained. Aliphatic aldehydes also provided α,α'-*bis*-(substituted-alkylidene)cycloalkanones in very good yields with minor amounts of α-(substituted-alkylidene)cycloalkanones. The catalytic performance of solid NaOH was examined. The molar ratio of NaOH was optimized. The catalytic effect of solid NaOH was also evaluated by comparing it with KOH, NaOAc, and NH_4_OAc and it turns out that 20 mol% of solid NaOH was good enough to catalyze the Claisen-Schmidt reactions of cycloalkanones with various substituted benzaldehydes. Additionally, the regioselectivity of the Claisen-Schmidt reaction of acetone with benzaldehyde was examined. Using the same method, we could synthesize the corresponding *bis*-benzylidene- and mono-benzylideneacetone separately in 98% and 96% yields, respectively.

## 1. Introduction

The Claisen-Schmidt reaction (crossed-aldol reaction) is a condensation reaction of aldehydes and carbonyl compounds leading to β-hydroxycarbonyl compounds and it has played an important role in synthetic organic chemistry [1,2,3,4,5,6]. Subsequent dehydration of the β-hydroxycarbonyl compounds afford α-alkylidene or α-arylidene compounds. Although studies on the Claisen-Schmidt reaction have been focused on α-alkylidene- and α-arylidene-carbonyl compounds, interest in α,α'-bisalkylidene- and α,α'-bisarylidene-carbonyl compounds is increasing. Particularly, α,α'-*bis*-(substituted-benzylidene)-cycloalkanones have been attracting much more attention, not only due to their intriguing biological activities such as antiangiogenic [7,8], quinine reductase inducer [9], arginine methyltransferase inhibitor [10], cytotoxicity [11,12], cholesterol-lowering activity [13], uses in agrochemicals, pharmaceuticals and perfumes [14], in *bis*-spiropyrrolidines [14,15,16], and as liquid crystalline polymer units [17], but also as important precursors for the synthesis of pyrimidine derivatives [18], 2,7-disubstituted tropones [19], and they are the synthetic intermediates of choice to functionalize the α, β-position during the total synthesis of natural products such as the cystodytins [20]. They have also been reported to possess drug resistance reversal properties [21,22].

The Claisen-Schmidt reactions of cycloalkanones leading to α,α'-*bis*-(benzylidene)cycloalkanones are classically catalyzed by strong acids [23,24] and more likely by base with or without solvent [25,26,27,28,29,30,31,32,33,34,35]. Various reagents have been introduced as the methodology was developed during last few decades, such as Cp_2_ZrH_2_ [36], Cp_2_TiPh_2_ [37], *bis*(*p*-methoxyphenyl)telluroxide (BMPTO) [38], RuCl_3_ [39], SmI_3_ [40,41], TiCl_3_(CF_3_SO_3_) [42], La^3+^-immobilized organic solid [43], KF-Al_2_O_3_ [44], Mg(HSO_4_)_2_ [45], FeCl_3_ [46], BF_3_·OEt_2_ [47], InCl_3_ [48], TMSCl/NaI [49], TMSCl/Pd-C [50], SOCl_2_ [51], Yb(OTf)_3_ [52], K_2_CO_3_/PEG-400 [53], molecular I_2_ [54], Cu(OTf)_2_ [55], silica chloride [56], silica-supported phosphorus pentoxide (P_2_O_5_/SiO_2_) or silicaphosphinoxide (silphox, [POCl_3-n_(SiO_2_)_n_]) as heterogeneous reagents [57], 1-methyl-3(2-(sulfooxy)ethyl)-1*H*-imidazol-3-ium chloride [58] and Et_3_N in the presence of LiClO_4_ [59]. The crossed-aldol condensation for the preparation of α,α'-*bis*(benzylidene) cycloalkanones is also catalyzed by animal bone meal (ABM) or Na/ABM [60], ionic liquid [61,62], sodium-modified-hydroxyapatite (Na-HAP) [63], micellar media [64], ethanolic KOH [65], 2,4,6-trichloro [1,3,5]triazine [66], polymer-supported sulphonic acid [67], lithium hydroxide monohydrate (LiOH•H_2_O) [68], and rare earth(III) perfluorooctane sulfonates [RE(OPf)_3_] [69]. However, most of the reactions suffer from reverse and/or side reactions [70,71,72] resulting in low yields of the desired products. Later, different complexes of metal (II) ions were used as catalysts to replace acids or bases but satisfactory yields were not obtained [73].

In our recent studies, we have synthesized α,α'-*bis*-(substituted-benzylidene)-cycloalkanones and substituted-benzylidene heteroaromatics using NaOAc [74] and NH_4_OAc [75] as catalysts. Due to the importance of the Claisen-Schmidt reaction in synthetic organic chemistry and of α,α'-*bis*-(substituted-benzylidene)-cycloalkanones as precursor for various natural products, we wish to report herein a facile solvent-free Claisen-Schmidt reaction using a grinding technique for the synthesis of α,α'-*bis*-(substituted-benzylidene)cycloalkanones, di- and/or mono- benzylidene acetone and benzylidene camphor using solid NaOH as catalyst.

## 2. Results and Discussion

### 2.1. α,α'-*bis*-(Substitutedbenzylidene)cycloalkanones

**Scheme 1:**
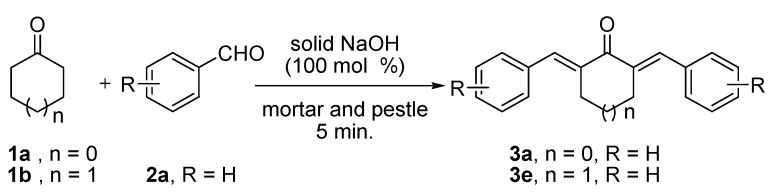
Solvent free Claisen-Schmidt reactions of **1a**/**1b** with **2a** in presence of NaOH (100 mol%) by grinding with a mortar and pestle for 5 min.

The Claisen-Schmidt reaction of cyclopentanone (**1a**, 10 mmol) or cyclohexanone (**1b**, 10 mmol) with benzaldehyde (**2a**, 20 mmol) in the presence of an equimolar amount of solid NaOH without any solvent after grinding with a mortar and pestle for 5 min. afforded the corresponding α,α'-*bis-*benzylidenecyclopentanone (**3a**) or α,α'-*bis*-benzylidenecyclohexanone (**3e**), both in 99% yield (Scheme 1).

We next examined the catalytic ability of NaOH by grinding cyclohexanone (**1b**, 10 mmol) with benzaldehyde (**2a**, 20 mmol) in presence of various molar ratios of NaOH (1–100) using the same procedure to afford the corresponding α,α'-*bis*-benzylidenecyclohexanone (**3e**). The results indicate that 20 mol% of NaOH gave satisfactory yield (98%) compared with a stoichiometric amount of NaOH. The results are summarized in Table 1. It should be noted that 10 mol% of NaOH gave 95% of the corresponding target compound **3e**, while 80 mol% and an equimolar amount of NaOH gave 99% yields.

**Table 1:** Claisen-Schmidt reactions of **1b** with **2a** using various mol% of NaOH by grinding in a mortar and pestle for 5 min.

Entry	NaOH (mol%) ^[a]^	Time (min.)	Yield ^[b]^
1	100	5	99
2	80	5	99
3	40	5	98
4	20	5	98
5	10	5	95
6	1	5	70

^[a]^ Relative to benzaldehyde; ^[b]^ Isolated yields, and were confirmed by proton NMR spectroscopy, which are not optimized.

Furthermore, we evaluated the effect of NaOH on the Claisen-Schmidt reaction of cyclohexanone (**1b**) with benzaldehyde (**2a**) over KOH and our previously reported catalysts NaOAc [74], and NH_4_OAc [75], (Table 2). The highest yield (98%) was achieved using 20 mol% of solid NaOH after grinding with a mortar and pestle for 5 minutes (entry 1, Table 2), while a slightly lower yield (85%) was obtained with 20 mol% of solid KOH (entry 2, Table 2). In addition, different experimental conditions were also applied to optimize the catalytic performance of solid NaOH by introducing solvent (EtOH) at room temperature as well as under refluxing conditions. When we stirred the reaction mixture with 20 mol% of NaOH in ethanol at room temperature for 24 hours (entry 3, Table 2), the product was obtained, but with low yield (40%), and much longer time (5 days) was required to obtain a 66% yield (entry 5, Table 2). After having no promising results with stirring at room temperature for 5 days, we then heated the reaction mixture of cyclohexanone (**1b**) and benzaldehyde (**2a**) to reflux for 8 hours with 20 mol% of NaOH in ethanol and this afforded the corresponding α,α'-*bis*-benzylidenecyclohexanone (**3e**) in 93% yield (entry 3, Table 2). Comparing all the results (entries 1 to 6, Table 2) with our previously reported methods (entries 7 and 8, Table 2) [74,75], we found that 20 mol% of solid NaOH and grinding with a mortar and pestle for 5 minutes is better than any other catalyst (such as KOH, NaOAc and NH_4_OAc tested) for the Claisen-Schmidt reaction of cyclohexanone (**1b**) with benzaldehyde (**2a**).

**Table 2:** Effect of the catalysts, solvents, temperature on the Claisen-Schmidt reactions of **1b** with **2a**.

Entry	Catalysts	Time	Yield ^[a] ^(%)
1	NaOH (20 mmol), mortar and pestle	5 min	98
2	KOH (20 mmol), mortar and pestle	5 min	85
3	NaOH (20 mmol), r.t, EtOH	24 h	40
4	NaOH (20 mmol), r.t, EtOH	96 h	60
5	NaOH (20 mmol), r.t, EtOH	5 d	66
6	NaOH (20 mmol), reflux , EtOH	8 h	93
7	NaOAc (20 mmol), AcOH, 120 °C	8 h	81–93[74]
8	NH_4_OAc (4 mmol), AcOH, 120 °C	8 h	83–95[75]
^[a]^ Isolated yields which are not optimized.			

Subsequently, we examined the scope and limitation of NaOH (20 mol%) as catalyst for the Claisen-Schmidt reaction of selected cycloalkanones (**1a** and **1b**) and a number of electronically modified aryl aldehydes **2a–h** employing grinding with a mortar and pestle for 5 minutes without any solvent to afford the corresponding α,α'-*bis*(substituted-benzylidene)cycloalkanones **3a–h**; the results are summarized in Table 3.

**Table 3:** The Claisen-Schmidt reaction of **1a–b** with **2a–h** in presence of solid NaOH (20 mol%) by grinding in a mortar and pestle for 5 min.

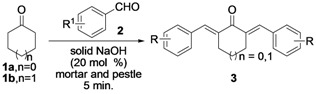				
Entry	R	n	Yield (%)	mp (°C) (lit.value) [references]
**3a**	H	0	98	188, (188–190) [ 74], (188–189) [38]
**3b**	2-Br	0	96	165, (163–165) [ 74], (162–163) [40]
**3c**	4-Me	0	98	184, (183–184) [ 40]
**3d**	4-OMe	0	98	211, (210–211) [ 74], (210–211) [38]
**3e**	H	1	98	119, (119–120) [ 74], (117–118) [38]
**3f**	2-NO_2_	1	98	159, (158–159) [ 74], (158–159) [74]
**3g**	3-Cl	1	97	104, (103–105) [ 74]
**3h**	4-Me	1	98	168, (165–167) [ 74], (170.1) [76]

**Scheme 2:**
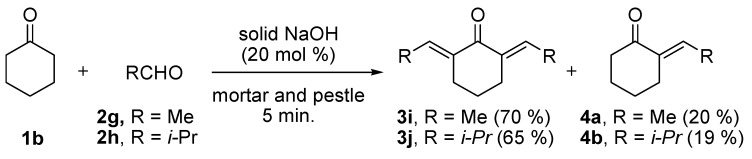
Solvent free Claisen-Schmidt reactions of 1b with **2g**/**2h** in the presence of NaOH (20 mol%) by grinding with a mortar and pestle for 5 min.

The electronic nature of the substituent on the benzene ring of compounds **2a–h** and the ring size of cycloalkanones (compounds **1a** and **1b**) did not affect the reaction and high yields (96–98%) were obtained for all the entries. We then attempted to prepare substituted-alkylidenecycloalkanones **3i** and **3j** from the reactions of cyclohexanone (**1b**) with acetaldehyde (**2g**) and *iso*-propanal (**2h**) using the optimized molar ratio of solid NaOH (20 mol%) to obtain the corresponding 2,6-*bis*-ethylidene-cyclohexanone (**3i**) and 2,6-*bis*-isobutylidenecyclohexanone (**3j**). It should be noted that we obtained a small amount of mono-substituted alkylidenecycloalkanone (**4a** and **4b**) along with the desired major products **3i** and **3j** (Scheme 2) and the conversion of **1b** was 100%.

### 2.2. Reaction of Acetone *(**5**)* with Benzaldehyde *(**2a**)*

**Scheme 3:**
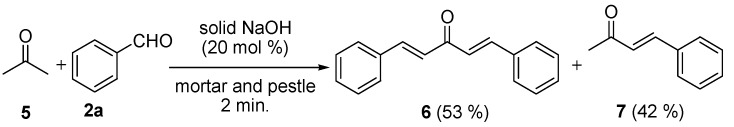
Claisen-Schmidt reactions of **5** with **2a** in the presence of NaOH (20 mol%) by grinding with a mortar and pestle for 5 min.

The Claisen-Schmidt reaction for acetone (**5**, 10 mmol) and benzaldehyde (**2a**, 20 mmol) was conducted for the preparation of *bis*-benzylidene acetone (**6**) using similar reaction conditions as shown in scheme 2, but the reaction time was only for 2 min. instead of 5 (Scheme 3). The reaction of **5** with **2a** gave a mixture of **6** and **7** in 53% and 42% yields, respectively.

Then we examined the regioselectivity of the reaction and the results are summarized in Table 4. Use of excess amount of **5** (>5 equiv.) resulted in mainly **7** (96%) with a trace amount of **6**, while on the other hand, when we used an excess of **2a** (>3 equiv.) *bis*-benzylideneacetone was obtained in 98% yield as a single product.

**Table 4:** The Claisen-Schmidt reaction of acetone (**5**) with benzaldehyde (**2a**) in presence of 20 mol% of solid NaOH by grinding in a mortar and pestle of 5 min.

Molar ratio		Conversion(%)	Yield ^[a] ^(%)	
5	2a		6	7
10 mmol	20 mmol	100 ^b^	53	42
Excess (>5 equiv.)	10 mmol	100 ^b^	trace	96
10 mmol	Excess (>3 equiv.)	100 ^c^	98	0

^[a]^ Isolated yields which are not optimized; ^[b]^ conversion of benzaldehyde; ^[c]^ conversion of acetone.

### 2.3. Reaction of 1,7,7-Trimethyl[2,2,1]hexan-2-one (Camphor, 8) with Substituted-benzaldehyde *(**2a*** and ***2c**)*

**Scheme 4:**
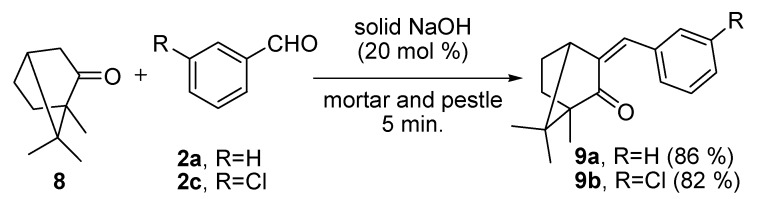
Claisen-Schmidt reactions of 8 with **2a/2c** in the presence of solid NaOH (20 mol%) by grinding with a mortar and pestle for 5 min.

We further examined the scope and limitations of this grinding technique for Claisen-Schmidt reactions by using substituted-benzaldehydes (compounds **2a** and **2c**) with 1,7,7-trimethyl[2,2,1]hexan-2-one (camphor, **8**) in the presence of 20 mol% NaOH. The reactions afforded the desired products benzylidene-1,7,7-trimethylbiyclo[2,2,1]hexan-2-one (**9a**) and 3-chloro-benzylidene-1,7,7-trimethylbicyclo[2,2,1]hexan-2-one (**9b**) in 86% and 82% yields, respectively (Scheme 4).

## 3. Experimental

### 3.1. General

Melting points were recorded on a Fisher–Jones melting point apparatus and are uncorrected. Nuclear magnetic resonance (NMR) spectra were recorded using a Bruker 250 spectrometer (250 MHz for ^1^H-NMR and 62.5 MHz for ^13^C-NMR) and are reported in parts per million (ppm) from the internal standard tetramethylsilane. Electrospray ionization (ESI) mass spectrometry (MS) experiments were performed on LCQ advantage-trap mass spectrometer (Thermo Finnigan, San Jose, CA, USA). 

### 3.2. Chemistry

#### 3.2.1. General Procedure for the Preparation of α,α'-*bis*-(Substituted-benzylidene)cycloalkanones **3a–h**

A mixture of cyclopentanone/cyclohexanone (**1a**/**1b**, 5.0 mmol), substituted benzaldehyde (**2a–h**, 10.0 mmol) and solid NaOH (20 mol%) was ground with a mortar and pestle at room temperature, under the hood for 5 minutes. The reaction mixture was poured into 2 N HCl, and the solid materials were collected and purified by flash chromatography on silica gel eluting with CH_2_Cl_2_-hexane (1:1) to give analytically pure α,α'-*bis*-(substituted-benzylidene)cycloalkanones **3a–h**.

*2,5-bis-(Benzylidene)cyclopentanone* (**3a**). Yellow solid (98%), mp. 188 °C, (188–190 °C) [24],(188–189 °C) [38].

*2,5-bis-(2-Bromobenzylidene)cyclopentanone* (**3b**). Yellow solid (96%). mp. 165 °C, (163–165 °C) [74], (162–163 °C) [40].

*2,5-bis-(4-Methylbenzylidene)cyclopentanone* (**3c**). Yellow solid (98%). mp. 184 °C, (183–184 °C) [40]. 

*2,5-bis-(4-Methoxylbenzylidene)cyclopentanone* (**3d**). Yellow solid (98%), mp. 211 °C, (210–211 °C) [74], (210–211 °C) [38]. 

*2,6-bis-(Benzylidene)cyclohexanone* (**3e**). Yellow solid (98%), mp. 119 °C, 119–120 °C [74],(117–118 °C) [38].

*2,6-bis(2-Nitrobenzylidene)cyclohexanone* (**3f**). Yellow needles (98%), mp. 159 °C, (158–159 °C) [74].

*2,6-bis(3-Chlorobenzylidene)cyclohexanone* (**3g**). Yellow needles (97%). mp. 104 °C, (103–105 °C) [74].

*2,6-bis(3-Methylbenzylidene)cyclohexanone* (**3h**). Yellow solid (98%), mp. 168 °C, (165–167 °C) [74] (170.1 °C) [76].

#### 3.2.2. General Procedure for the Preparation of **3i**, **3j** and **4**

2,6-*bis*-Alkylcycloalkanones **3i** and **3j** and *α*-(mono)alkylcycloalkanones **4a** and **4b** were obtained as solid/oily materials following the procedure adopted for **3a–h** from a mixture of cyclohexanone (**1b**, 5.0 mmol) and acetaldehyde (**2g**) or *iso*-propanal (**2h**) (10.0 mmol) and were purified by flash chromatography on silica gel eluting with CH_2_Cl_2_-hexane (1:1).

*2,6-bis-Ethylidene-cyclohexanone* (**3i**). Colorless oil (70%): bp. 130 °C (0.5 mm Hg) [75].

2,6-bis-Isobutylidene-cyclohexanone (**3j**). Colorless oil (65%) [68,75].

*(*E*)-2-Ethylidenecyclohexanone* (**4a**). Colorless oil (20%), bp. 76–80 °C (14 mm Hg) [77], bp. 87–89 °C (18 mm Hg) [75].

*2-Isobutylidenecyclohexanone* (**4b**). Colorless oil (19%) [78].

#### 3.2.3. Procedure for the Preparation of (1*E*,4*E*)-1,5-Diphenylpenta-1,4-dien-3-one (**6**) and (*E*)-4-Phenylbut-3-en-2-one (**7**)

(1*E*,4*E*)-1,5-diphenylpenta-1,4-dien-3-one (**6**) and (*E*)-4-phenylbut-3-en-2-one (**7**) were obtained following the procedure adopted for **3a–h** from mixture of acetone (**5**, 5.0 mmol) and benzaldehyde (**2a**, 10.0 mmol). The oily material was collected and purified by flash chromatography on silica gel.

*(1*E*,4*E*)-1,5-Diphenylpenta-1,4-dien-3-one* (**6**). Yellow solid: mp 109–111 °C, (107 °C) [79], (112 °C). [80] ^1^H NMR (CDCl_3_, 250 MHz): δ 7.73 (d*, J* = 15.9 Hz, 2H), 7.61–7.59 (m, 4H), 7.41–7.38 (m, 6H), 7.07 (d, *J* = 15.9 Hz, 2H).

*(*E*)-4-Phenylbut-3-en-2-one* (**7**). Low melting solid mp 39–42 °C) ^1^H-NMR (CDCl_3_, 250 MHz): 7.55–7.51 (m, 2H), δ 7.50 (d*, J* = 16.4 Hz, 1H), 7.39–7.36 (m, 3H), 6.70 (d, *J* = 16.2 Hz, 1H), 2.37 (s, 3H). ^13^C-NMR (CDCl_3_, 62.5 MHz): *δ* 198.5, 143.47, 134.31, 130.5, 128.93, 128.22, 127.07, 27.49. 

#### 3.2.4. Preparation of (*E*)-3-Substitutedbenzylidene-1,7,7-trimethylbicyclo[2.2.1]heptan-2-one (**9**)

(*E*)-3-Benzylidene-1,7,7-trimethylbicyclo[2.2.1]heptan-2-one (**9a**) and *(*E)-3-(3-chloro-benzylidene)-1,7,7-trimethylbicyclo[2.2.1]heptan-2-one (**9b**) were obtained following the procedure adopted for **3a–h** from a mixture of 1,7,7-trimethyl[2,2,1]hexan-2-one (camphor, **8**, 5.0 mmol) and substituted benzaldehyde (**2a/2c**, 5.0 mmol). The oily material was collected and purified by flash chromatography on silica gel eluting with EtOAc-hexane (1:19).

*(*E*)-3-Benzylidene-1,7,7-trimethylbicyclo[2.2.1]heptan-2-one* (**9a**). White solid (86%). mp. 74 °C, (71–73 °C) [81], (74–75 °C) [82]; ^1^H-NMR (CDCl_3_, 250 MHz): δ 7.48–7.28 (m, 5H), 7.22 (s, 1H), 3.09 (d, *J* = 4.1 Hz, 1H), 2.21–2.12 (m, 1H), 1.96–1.71 (m, 1H), 1.63–1.46 (m, 2H), 1.01 (s, 3H), 0.98 (s, 3H), 0.78 (s, 3H). ^13^C-NMR (CDCl_3_, 62.5 MHz): *δ* 208.33, 142.09, 135.67, 129.76, 128.68, 128.63, 127.52, 57.11, 49.17, 46.70, 30.67, 25.94, 20.57, 18.32, 9.30. 

*(*E*)-3-(3-chlorobenzylidene)-1,7,7-trimethylbicyclo[2.2.1]heptan-2-one* (**9b**). White solid (82%). ^1^H-NMR (CDCl_3_, 250 MHz): δ 8.07 (s, 1H), 7.97 (d, *J* = 7.8 Hz, 1H), 7.55 (d, *J* = 7.8 Hz, 1H), 7.40 (t, *J* = 7.8 Hz, 1H), 7.35 (s, 1H), 2.40–2.23 (m, 1H), 2.07–1.95 (m, 1H), 1.85–1.78 (m, 1H), 1.68–1.62 (m, 1H), 1.40–1.30 (m, 1H), 0.93 (s, 3H), 0.98 (s, 3H), 0.81(s, 3H).

## 4. Conclusions

A facile solvent-free Claisen-Schmidt reaction between cyclopentanone (**1a**)/cyclohexanonoe (**1b**) and different substituted benzaldehydes **2a–h** catalyzed by solid NaOH (20 mol%) by applying a grinding technique using a mortar and pestle for 5 minutes was performed, resulting in excellent yields (96–98%) of the corresponding α,α'-*bis*(substituted-benzylidene)cyclo-alkanones **3a–h**. The Claisen-Schmidt reaction using NaOH was optimized and it turned out that 20 mol% of NaOH is sufficient to perform the reactions. The catalytic effect was also examined and we found 20 mol% of solid NaOH is better than any other catalyst tested such as KOH, NaOAc and NH_4_OAc, for the Claisen-Schmidt reaction under solvent free condition. Beside aryl aldehydes, alkyl aldehydes were also converted to their corresponding *bis*-alkylidenecycloalkanones along with a little mono alkylidene cycloalkanone by the method in question. Additionally, we examined the regioselectivity of the Claisen-Schmidt reaction by reacting acetone (**5**) with benzaldehyde (**2a**) leading to give the corresponding *bis*-benzylideneacetone in 98% yield using the same method.
